# Effect of pupil dilation on intraocular pressure in preterm and term infants

**DOI:** 10.5935/0004-2749.20220058

**Published:** 2025-08-22

**Authors:** Ayşe Çiçek, Damla Ergintürk Acar, Osman Baştuğ, Hatice Birgin, Mustafa Ataş, Bedirhan Alabay, Necati Duru

**Affiliations:** 1 Department of Ophthalmology, Kayseri Education and Research Hospital, Kayseri, Turkey; 2 Department of Ophthalmology, Zekai Tahir Burak Women’s Health Training and Research Hospital, Ankara, Turkey; 3 Department of Pediatrics, Kayseri Education and Research Hospital, Kayseri, Turkey

**Keywords:** Infant, Infant, newborn, Infant, premature, Intraocular pressure, Pupil, Phenylephrine, Tropicamide, Dilatation, Lactente, Recém-nascido, Recém-nascido prematuro, Pressão Intraocular, Pupila, Fenilefrina, Tropicamida, Dilatação

## Abstract

**Purpose:**

To evaluate the effect of pupil dilation on intraocular pressure in preterm and term newborns.

**Methods:**

This prospective study involved 55 eyes of 28 preterm infants and 38 eyes of 20 term infants. The infants were divided into two groups according to their gestational ages at birth as follows: preterm group, <37 weeks and term group, ≥37 weeks. Pupil dilation was attained with tropicamide 0.5% and phenylephrine 2.5%. Intraocular pressure measurements were performed with Icare PRO (Icare Finland Oy, Helsinki, Finland) before and after pupil dilation. A paired *t* test was used to compare the measurements before and after pupil dilation.

**Results:**

The mean intraocular pressure change was -1.04 ± 3.03 mmHg (6.20/-11.40 mmHg) in the preterm group and -0.39 ± 2.81 mmHg (4.60/-9.70 mmHg) in the term group. A statistically significant difference in intraocular pressure was observed only in the preterm group after pupil dilation (p=0.01).

**Conclusion:**

An unexpected alteration in intraocular pressure in newborns may occur after pupil dilation, especially in preterm infants.

## Abstract (pt)

**Objetivo:**

Avaliar o efeito da dilatação da pupila sobre a pressão intraocular em recém-nascidos pré-termo e a termo.

**Métodos:**

Este estudo prospectivo envolveu 55 olhos de 28 bebês pré-termo e 38 olhos de 20 bebês a termo. Os bebês foram divididos em dois grupos, pré-termo e a termo, de acordo com a idade gestacional ao nascimento: grupo pré-termo <37 semanas; grupo a termo ≥37 semanas. A dilatação da pupila foi feita com tropicamida 0,5% e fenilefrina 2,5%. As medições da pressão intraocular foram realizadas com Icare PRO (Icare Finland Oy, Helsinki, Finlândia) antes e depois da dilatação da pupila. O teste t pareado foi usado para comparar as medidas antes e depois da dilatação da pupila.

**Resultados:**

A alteração média da pressão intraocular foi de -1,04 ± 3,03 mmHg (+6,20/-11,40 mmHg) no grupo pré-termo e -0,39 ± 2,81 mmHg (+4,60/-9,70 mmHg) no grupo a termo. Uma diferença estatisticamente significativa na pressão intraocular foi observada apenas no grupo pré-termo após a dilatação da pupila (p=0,01).

**Conclusão:**

Após a dilatação da pupila, pode ocorrer alteração inesperada da pressão intraocular em recém-nascidos, principalmente em bebês pré-termo.

## INTRODUCTION

Pharmacological dilation of the pupil is necessary for examining the posterior segment of the eyes in children and adults. The most frequently used medications for pupil dilation in daily practice are tropicamide, phenylephrine, and cyclopentolate. Increased intraocular pressure (IOP) after pupil dilation has been reported in open-angle glaucoma^(1-7)^. However, some studies have shown contradicting results about the effect of pupil dilation on IOP in healthy subjects^(8-11)^. IOP spikes after pupil dilation were reported in some studies, which necessitates caution during the procedure.

Pupil dilation is a key step in screening newborns, mainly for retinopathy of prematurity (ROP) and other diseases. The drops used for pupil dilation in newborns are similar to those used in adults but at lower concentrations. The use of tropicamide 0.5%, phenylephrine 2.5%, and cyclopentolate 0.5% has been recommended in the screening for ROP in Turkey by the Turkish Neonatology Association and many countries worldwide^(12)^. However, we prefer to use the combination of tropicamide 0.5% and phenylephrine 2.5% in ROP screening and examination of term newborns to avoid the systemic side effects of cyclopentolate^(13)^. To the best of our knowledge, no study has investigated the effect of pupillary dilation with tropicamide 0.5% and phenylephrine 2.5% on IOP in preterm and term infants. This study aimed to evaluate the effect of pupil dilation on IOP in preterm and term newborns.

## METHODS

This prospective study was conducted in the ophthalmology department of our institute. It adhered to the tenets of the Declaration of Helsinki. Oral and written informed consent was received from the parents of all the newborns included in the study. The study was approved by the institutional review board of our university hospital.

Regardless of gestational age (GA), all infants are referred for examination to an ophthalmologist in the first month of birth in accordance with the protocol of the National Screening Program in Turkey. In addition, preterm infants with GAs <32 weeks are referred to the ophthalmology department for ROP screening. Preterm and term infants referred for ophthalmological examination with pupil dilation were enrolled in the study. Infants with any ocular disease (e.g., ROP, cataract, coloboma of iris, lens, or optic nerve), systemic disease (e.g., respiratory distress syndrome, necrotizing enterocolitis, and infectious disease), history of topical or systemic drug use, and inadaptability (inability to calm down or crying continuously during the examination) to IOP measurement were excluded from the study to avoid incorrect high measurements caused by the Valsalva maneuver while crying.

The infants were divided into two groups according to their GAs at birth as follows: preterm group, <37 weeks and term group, ≥37 weeks. After the administration of one drop of proparacaine hydrochloride 0.5% ophthalmic solution (Alcaine, Alcon, Fort Worth, TX, USA), the eyelid speculum was placed. IOP measurements were performed with Icare PRO (Icare Finland Oy, Helsinki, Finland) after ensuring that the infant had calmed down. Comforters and a few drops of 20% dextrose solution were used to help the infants relax in some cases. All the measurements were performed at the same time of the day by the same experienced ophthalmologist (A.Ç.). Five consecutive measurements were made in each session, the average of which was used for the analysis. After the first measurements, drops of tropicamide 0.5% and phenylephrine 2.5% were applied twice per eye with an interval of 5 minutes between each drop. The interval between the last drop and the examination was at least 40 minutes. IOP measurement after pupil dilation was executed immediately before the examination. Infants with a pupil diameter of <6.0 mm after dilation and a diagnosis of any ocular disease, including ROP, were excluded from the study.

### Statistical analyses

SPSS version 20.0 for Windows (IBM, Chicago, IL) was used for the analysis. The Shapiro-Wilk test was used for the assessment of data distribution. A paired *t* test was used to compare the measurements before and after pupil dilation. The correlation between the measurements before and after pupil dilation was evaluated by calculating the Pearson correlation coefficient. A p value <0.05 was assessed to be statistically significant. The Pearson correlation coefficient was assessed as follows: <0.29 = low correlation, 0.30-0.49 = medium correlation, and 0.50-1.00 = strong correlation.

The minimum sample size with a 95% confidence in terval and 90% power to detect the difference of >2 mmHg (clinically significant difference consistent with previous studies)^(3,6)^ was decided as at least 42 eyes for the preterm group and 34 eyes for the term group.

## RESULTS

A total of 55 eyes of 28 preterm infants and 38 eyes of 20 term infants were involved in the study. The mean (range) GA at birth and postconceptual age at examination were respectively 32.20 ± 2.57 weeks (27-36 weeks) and 38.28 ± 5.96 weeks (30-52 weeks) in the preterm group and 39.48 ± 1.25 weeks (37-41 weeks) and 44.24 ± 4.32 weeks (38-56 weeks) in the term group. The demographic characteristics and IOP measurements before dilation are summarized in table 1.

**Table 1:** Demographic Characteristics and Intraocular Pressure (IOP) Values of the Two Infant Groups

	Preterm group (n=55 eyes of 28 infants)	Term group (n=38 eyes of 20 infants)
Sex, *%* (male/female)	42/58 (12/16)	40/60 (8/12)
Gestational age, *weeks* (mean ± SD)	32.20 ± 2.57	39.48 ± 1.25
Postconceptional age, *weeks* (mean ± SD)	38.28 ± 5.96	44.24 ± 4.32
IOP before pupil dilation, mmHg	12.40 ± 3.35	15.73 ± 3.78
IOP after pupil dilation, mmHg	11.28 ± 3.00	15.16 ± 3.79

SD= Standard deviation.

In the preterm and term groups, the mean (range) IOP values were respectively 12.40 ± 3.35 mmHg (7.00-21.70 mmHg) and 15.73 ± 3.78 mmHg (8.9023.90 mmHg) before pupil dilation and 11.28 ± 3.00 mmHg (6.20-21.30 mmHg) and 15.16 ± 3.79 mmHg (7.0021.80 mmHg) after dilation. A statistically significant difference in the IOP was observed in the preterm group after pupil dilation, whereas no significant difference was found in the term group (p=0.01 vs p=0.39). In the preterm and term groups, the mean IOP changes were -1.04 ± 3.03 and 0.39 ± 2.81 mmHg; maximum IOP increases after dilation, 6.20 mmHg (from 7.30 to 13.50 mmHg) and 4.60 mmHg (from 14.00 to 18.60 mmHg); and maximum IOP decreases after dilation, 11.40 mmHg (from 21.00 to 9.60 mmHg) and 9.70 mmHg (from 23.90 to 14.20 mmHg), respectively. Of the eyes in the preterm and term groups, 7% (4/56) and 21% (8/38) had an IOP increase of >2 mmHg and 1% (1/56) and none had an IOP increase of >5 mmHg after dilation, respectively; 30% (17/56) and 21% (8/38) had an IOP decrease of >2 mmHg and 8% (5/56) and 7% (3/38) had an IOP decrease of >5 mmHg after dilation; and the maximum and minimum IOP values after dilation were 21.30 and 6.20 mmHg and 21.80 and 7.00 mmHg, respectively. A strong correlation was found between the IOP values before and after dilation in both groups (r=0.69 and p≤0.001 for the preterm group and r=0.73 and p≤0.001 for the term group).

## DISCUSSION

We determined a statistically significant IOP decrea se in the preterm infants in our study. Some studies have reported contradicting results about the effect of pupillary dilation on IOP in healthy adults. Atalay et al.^(8)^ investigated the effect of pupil dilation with tropicamide 1% and phenylephrine HCL 10% on IOP in patients with and patients without pseudoexfoliation. They detected a statistically significant difference only in the control group involving healthy volunteers. The mean IOP decrease was -2.89 ± 0.72 mmHg in the control group (p=0.001). Conversely, Kim et al.^(10)^ found an IOP increase (mean, 1.85 ± 2.01 mmHg; p=0.002) after pupil dilation with tropicamide 1% and phenylephrine 2.5% in healthy adults. In another study^(9)^, the mean IOP was reduced by 1.1 ± 2.5 mmHg in the right eye and 0.7 ± 2.3 mmHg in the left eye after pupillary dilation with tropicamide 1% and phenylephrine 2.5% in healthy subjects. Moreover, they observed a high variability between the individual responses to dilation (range, 5 to -6 mmHg).

We investigated the effect of pupillary dilation with tropicamide 0.5% and phenylephrine 2.5% on IOP in preterm and term infants. This is the first study in this respect. Although many studies have evaluated IOP in preterm and term infants, no study has investigated the change in IOP after pupil dilation in either preterm or term infants. The mean (range) IOP values before dilation were 12.40 ± 3.35 mmHg (7.00-21.70 mmHg) and 15.73 ± 3.78 mmHg (10.50-23.90 mmHg) in the preterm and term groups, respectively (p<0.001). These values were lower than the measurements in other studies. The mean IOP values in some studies ranged from 14.1 ± 1.9 mmHg^(14)^ to 18.9 ± 3.7 mmHg^(15)^ in preterm infants and from 15.99 ± 2.79 mmHg^(16)^ to 17 ± 2.6 mmHg^(16)^ in term infants^(17-20)^. The IOP measurement was performed using Tono-Pen XL in all the studies. However, we used Icare PRO in the IOP measurement in this study. McKee et al.^(21)^ reported that the IOP measured using Tono-Pen in the supine position was 2 mmHg higher than that measured using Icare PRO in anesthetized children. In addition, this difference was greater in the eyes with frank corneal edema.

Nakamura et al.^(22)^ compared the Icare rebound tonometer with the Goldmann applanation, Tono-Pen XL, and noncontact tonometers. They found that the mean difference in IOP between the Icare and Tono-Pen XL measurements was 0.00 ± 4.78 mmHg. Tono-Pen XL is an applanation tonometer, whereas Icare PRO is a rebound tonometer. Measurement with Tono-Pen XL is mainly dependent on the central corneal thickness (CCT). Although measurement with Icare PRO is also affected by the viscoelastic properties of the cornea, it is less affected by CCT than Tono-Pen XL^(23)^. In addition, Acar et al.^(24)^ associated increased IOP values in premature infants with higher CCT values in their prospective longitudinal study. The results of the previous studies could explain the lower IOP values in our study.

In this study, the IOP measurements tended to decrease mainly in the preterm and term groups after pupil dilation. This result was similar to those of Atalay et al.^(8)^ and Qian et al.^(9)^. The IOP change after dilation was highly variable in both groups, ranging from 6.20 to -11.40 mmHg in the preterm group and from 4.60 to -9.70 mmHg in the term group. Tsai et al.^(11)^ investigated the IOP change after mydriasis with tropicamide 1% in children. They reported a high interindividual variation in IOP change, ranging from 8.0 to -8.0 mmHg, although the difference between the IOP values before and after mydriasis was not statistically significant. Qian et al.^(9)^ also reported a high variability but narrower range of IOP change than that in our study (ranging from 5 to -6 mmHg). IOP values >19.02 mmHg were not observed in either group. In terms of avoiding glaucomatous damage, pupillary dilation seems reliable for preterm and term infants. Nevertheless, the IOP values <5 mmHg measured in both groups were worrisome in terms of complications related to ocular hypotony. The IOP level that could cause damage to the ocular structure was found to be ≤5 mmHg for adults in a previous study^(25)^. However, no study has observed ocular hypotony in preterm and term infants. Follow-up of IOP in preterm and term infants after pupil dilation will be beneficial to prevent the damage caused by ocular hypotony. IOP spikes after dilation were not observed in any of the subjects. The correlation between the IOPs before and after dilation was strong in both groups (*r*=0.69 and p≤0.001 for the preterm group; r=0.73 and p≤0.001 for the term group). This result indicates that eyes with lower IOP values before dilation have a higher risk of developing ocular hypotony.

The pathophysiology of hypertony or hypotony after pupil dilation is unclear. Only suggestions about the mechanism of IOP change after pupillary dilation have been reported. Obstruction of the trabecular meshwork because of pigment dispersion from the iris and decreased aqueous outflow related to ciliary muscle paralysis are the most popular suggested mechanisms for hypertony^(2-5)^. Hypertony after dilation was less commonly observed in this study because of the differences in the structures of the iris, trabeculum, and anterior chamber angle between infants and adults. The iris and ciliary body are inserted in the scleral spur at birth. After the first year of life, posterior placement forming an angle recession occurs. The uveal and trabecular meshwork are less pigmented at birth than in adulthood. The peripheral iris is flatter and thinner in infants than in adults, and iris processes are rarely observed at birth. Increased uveoscleral outflow because of decreased muscle tone in the ciliary body is one of the postulated mechanisms of hypotony in adults^(26)^. The sclera is less strong and more elastic in infants than in adults. In addition, the infant sclera has almost half the thickness of the adult sclera. This configuration facilitates the uveoscleral outflow of the aqueous humor. All these arrangements make the eyes of infants prone to hypotony rather than hypertony after dilation.

One of the limitations of this study is the use of only tropicamide 0.5% and phenylephrine 2.5% at a single concentration. Further studies are needed to investigate the impact of other mydriatic agents such as cyclopentolate on IOP in infants. Another limitation of the study is the short follow-up time. Delayed hypotony or hypertony could not be assessed in this study, and the duration of the effect of dilation on IOP could not be determined. The absence of other ocular assessments such as gonioscopy or the measurement of corneal thickness is another limitation of this study. All IOP measurements (before and after pupil dilation) were performed after the placement of an eyelid speculum. This means that the placement of an eyelid speculum does not affect the difference in IOP caused by pupil dilation. The IOP measurement was not performed before the placement of the eyelid speculum. Nevertheless, this is not a limitation of our study because our purpose was to assess the effect of pupil dilation on IOP.

In conclusion, the IOP change after pupil dilation with tropicamide 0.5% and phenylephrine 2.5% was statistically significant only in the preterm group (preterm vs term group: -1.04 ± 3.03 mmHg vs -0.39 ± 2.81 mmHg). The variations in IOP change between the subjects (range, 6.20 to -11.40 mmHg in the preterm group and 4.60 to -9.70 mmHg in the term group) were also distinct. Unexpected alterations in the IOP in newborns may occur after pupil dilation, especially in preterm infants. Follow-up IOP measurement after pupil dilation seems to be essential in all infants, especially those born preterm.
